# The transcriptional factor Clr-5 is involved in cellulose degradation through regulation of amino acid metabolism in *Neurospora crassa*

**DOI:** 10.1186/s12896-023-00823-4

**Published:** 2023-11-29

**Authors:** Fanglei Xue, Zhen Zhao, Shuying Gu, Meixin Chen, Jing Xu, Xuegang Luo, Jingen Li, Chaoguang Tian

**Affiliations:** 1 College of Biotechnology, Tianjin University of Science and Technology, Tianjin, 300457 China; 2 Key Laboratory of Engineering Biology for Low-Carbon Manufacturing, Tianjin Institute of Industrial Biotechnology, Chinese Academy of Sciences, Tianjin, 300308 China; 3 National Technology Innovation Center of Synthetic Biology, Tianjin, 300308 China

**Keywords:** *Neurospora crassa*, Transcription factor, Cellulase production, Amino acid metabolism, Clr-5

## Abstract

**Background:**

Filamentous fungi are efficient degraders of plant biomass and the primary producers of commercial cellulolytic enzymes. While the transcriptional regulation mechanisms of cellulases have been continuously explored in lignocellulolytic fungi, the induction of cellulase production remains a complex multifactorial system, with several aspects still largely elusive.

**Results:**

In this study, we identified a Zn_2_Cys_6_ transcription factor, designated as Clr-5, which regulates the expression of cellulase genes by influencing amino acid metabolism in *Neurospora crassa* during growth on cellulose. The deletion of *clr-5* caused a significant decrease in secreted protein and cellulolytic enzyme activity of *N. crassa*, which was partially alleviated by supplementing with yeast extract. Transcriptomic profiling revealed downregulation of not only the genes encoding main cellulases but also those related to nitrogen metabolism after disruption of Clr-5 under Avicel condition. Clr-5 played a crucial role in the utilization of multiple amino acids, especially leucine and histidine. When using leucine or histidine as the sole nitrogen source, the Δ*clr-5* mutant showed significant growth defects on both glucose and Avicel media. Comparative transcriptomic analysis revealed that the transcript levels of most genes encoding carbohydrate-active enzymes and those involved in the catabolism and uptake of histidine, branched-chain amino acids, and aromatic amino acids, were remarkably reduced in strain Δ*clr-5*, compared with the wild-type *N. crassa* when grown in Avicel medium with leucine or histidine as the sole nitrogen source. These findings underscore the important role of amino acid metabolism in the regulation of cellulase production in N. crassa. Furthermore, the function of Clr-5 in regulating cellulose degradation is conserved among ascomycete fungi.

**Conclusions:**

These findings regarding the novel transcription factor Clr-5 enhance our comprehension of the regulatory connections between amino acid metabolism and cellulase production, offering fresh prospects for the development of fungal cell factories dedicated to cellulolytic enzyme production in bio-refineries.

**Supplementary Information:**

The online version contains supplementary material available at 10.1186/s12896-023-00823-4.

## Introduction

Plant biomass represents an abundant and renewable energy source that can be used in biorefinery processes [1, 2]. Filamentous fungi are recognized as primary contributors to the degradation of plant biomass due to their capability to produce enzymes that can effectively break down complex polysaccharides. The industrial production of fungal cellulases is poised to play a pivotal role in the synthesis of biochemicals and biofuels [3]. Notable examples of well-studied filamentous fungi in this context include *Neurospora crassa* [4–8], *Myceliophthora thermophila* [9–11], *Trichoderma reesei* [12, 13], and various *Aspergillus* species [14].

The degradation of cellulose in filamentous fungi relies on the coordinated action of a suite of enzymes. These enzymes are activated by plant-derived biopolymers or their derivatives and are predominantly regulated at the transcriptional level by specific fungal transcription factors [8]. The first transcription factor identified as being involved in cellulosic biomass degradation was XlnR, which mediates xylose-triggered induction of the expression of genes encoding xylanolytic and cellulolytic enzymes in *Aspergillus* [15]. Since then, a number of transcription regulators broadly involved in cellulose degradation have been discovered, including the transcriptional activators CLR-1/2, Hac1, Prd-1, Vib-1, and Rac-1 and the transcriptional repressors Cre-1, AmyR, and Ace1 [16–19], all of which participate in the regulation of cellulolytic and/or hemicellulolytic enzyme production. For example, XLR-1 in *N. crassa* and its ortholog XlnR in *Fusarium graminearum* play pivotal roles in xylanase production [8, 20], while the orthologs of XLR1 in *T. reesei* and *Aspergillus niger* are indispensable for the synthesis of both hemicellulase and cellulase [21–23]. In *N. crassa*, CLR-1 and CLR-2 are essential regulators of cellulase production [24], as do CLR-2 orthologs in *Aspergillus nidulans* (ClrB) and *Aspergillus oryzae* (ManR) [16, 25]. The transcription factor Hac1, which is involved in the unfolded protein response pathway, affected cellulase gene expression in *N. crassa* and *M. thermophila* [26, 27]. In *T. reesei*, cellulase production and protein secretion were found to be significantly improved in a line overexpressing regulatory gene *vib-1* [28]. The deletion of the carbon catabolite repressor, Cre-1 in *N. crassa*, led to increased cellulolytic enzyme activity and upregulated the transcription of genes responsible for encoding cellulolytic enzymes when grown on Avicel [19]. Moreover, overexpression of AmyR, a key regulator of amylolytic enzymes, resulted in a 30% increase in amylase activity in *M. thermophila* [29].

Several studies have explored the potential regulatory links between nitrogen metabolism and polysaccharide degradation/utilization. Microorganisms have evolved intricate transcriptional networks to prioritize the utilization of available nutrients [30]. Some transcription factors regulate not only extracellular protease production, but also nitrogen metabolism. For example, AreA, apart from regulating extracellular protease production, as observed in *A. nidulans* and *N. crassa* [31, 32], also controls the expression of genes encoding cellulases [33]. In *N. crassa*, Amn-1 is required for the utilization of non-preferred nitrogen sources (proline, branched-chain amino acids, and aromatic amino acids) and plays a role in regulating genes involved mannose and mannan utilization, suggesting that nitrogen metabolism and carbon metabolism are integrated to some extent [30]. The transcription factor TAM1, a homolog of TamA that regulates nitrogen metabolism in *A. nidulans* [34], not only governs ammonium utilization but also influences cellulase gene expression in *T. reesei* [35].

The filamentous fungus *N. crassa* possesses the capacity to secrete a vast array of enzymes crucial for lignocellulose utilization. Extensive research in genetics, biochemistry, and molecular biology has been conducted on this fungus [36], making it an ideal model system for elucidating the mechanisms governing the expression and regulation of genes encoding cellulases. In nature, *N. crassa* colonizes freshly burnt plant biomass and shows robust growth on lignocellulose [3]. Unlike many other filamentous fungi, *N. crassa* boasts a nearly complete genome deletion strain collection. Its cellulolytic potential has been recognized for decades, and its genome encodes a comparable number of glycoside hydrolases to that of *T. reesei*, the primary industrial source of enzymes for biomass depolymerization [5]. When grown on straw residues derived from various crops, such as barley, corn, rice, soybean, and wheat, a large proportion of the genes encoding CAZymes are induced in *N. crassa* [6]. The *N. crassa* genome also hosts 14 genes encoding lytic polysaccharide monooxygenases (LPMOs), classified within the Carbohydrate-Active Enzyme (CAZyme) family AA9 [37]. In *N. crassa*, the Zn_2_Cys_6_ transcription factor plays a crucial role in coordinating the production of cellulolytic enzymes. Several regulators, including CLR-1, CLR-2, CLR-4, XLR-1, CRE-1, COL-26, and VIB1, have been studied for their involvement in cellulase production [19, 24, 38–40]. In this study, we identified a Zn_2_Cys_6_ transcription factor, Clr-5, which regulates the expression of genes encoding cellulases in *N. crassa* during growth on cellulose. The deletion of *clr-5* in *N. crassa* led to a marked reduction in secreted protein levels and (hemi)cellulolytic enzyme activity. The growth phenotypes of *N.crassa* Δ*clr-5* on Avicel medium could be partially restored by the addition of yeast extract. Clr-5 played an important role in amino acid metabolism. When leucine or histidine was supplied as the sole nitrogen source, Δ*clr-5* showed severe growth defects and down-regulated expression of most genes encoding CAZymes and those involved in the uptake and metabolism of multiple amino acids. This finding may be exploited to engineer filamentous fungal cell factories capable of producing cellulase or biofuels from plant biomass.

## Results and discussion

### Identification of the Zn_2_Cys_6_ transcription factor Clr-5 that regulates the cellulase production in *N. crassa*

**Fig. 1:**
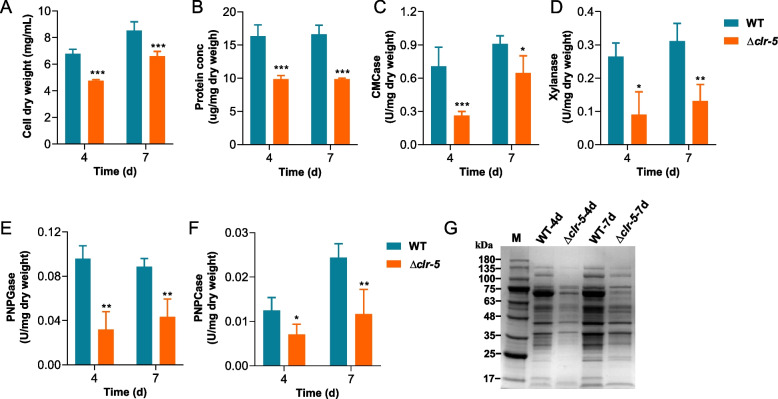
Protein production and enzyme activity phenotypes of the mutant Δ*clr-5* on Avicel. Biomass accumulation (**A**), total extracellular protein concentration (**B**), endo-glucanase (CMCase) activity (**C**), xylanase activity (**D**), β-glucosidase (PNPGase) activity (**E**), exo-cellulase (PNPCase) activity (**F**) in the supernatants of cultures of wild-type (WT) and Δ*clr-5* after 7 d or 4 d of growth in 2% Avicel medium. **G** SDS-PAGE analysis of the proteins secreted by *N. crassa* strains in Avicel medium. Error bars indicate the standard deviations from at least three biological replicates

In our quest to identify novel regulators of cellulase production, we systematically screened single-gene mutants of *N. crassa*. Specifically, we focused on those with the deletion of the gene encoding Zn_2_Cys_6_ transcription factors. Through this approach, we pinpointed a *N. crassa* strain deficient in the gene encoding the transcription factor NCU05383. Notably, this strain displayed a pronounced defect in cellulase production. Therefore, we designated NCU05383 as Clr-5 (cellulose degradation regulator 5). After growth on Avicel medium for 7 d, the mutant Δ*clr-5* exhibited a significant decrease in secreted protein (~ 54%), endo-glucanase activity (~ 45%), endo − 1,4 − β − xylanase activity (~ 67%), β-glucosidase activity (~ 62%), and exocellulase activity (~ 60%) compared with those of *N. crassa* wild-type (WT) strain (Fig. 1B-F). This result highlighted the important role of Clr-5 in cellulase production in *N. crassa*. Protein profiling of the extracellular secretome via SDS-PAGE confirmed a notable impairment in cellulase production in the Δ*clr-5* strain (Fig. 1G). Furthermore, the mycelium dry weight of Δ*clr-5* was 35.4% lower than that of the WT strain (Fig. 1A). However, Δ*clr-5* accumulated a comparable amount of biomass to that of the WT strain when grown on glucose (Additional file 1: Fig. S1), providing evidence that Clr-5 is not involved in fungal growth and thereby confirm the effect of Clr-5 on cellulase production.

**Fig. 2:**
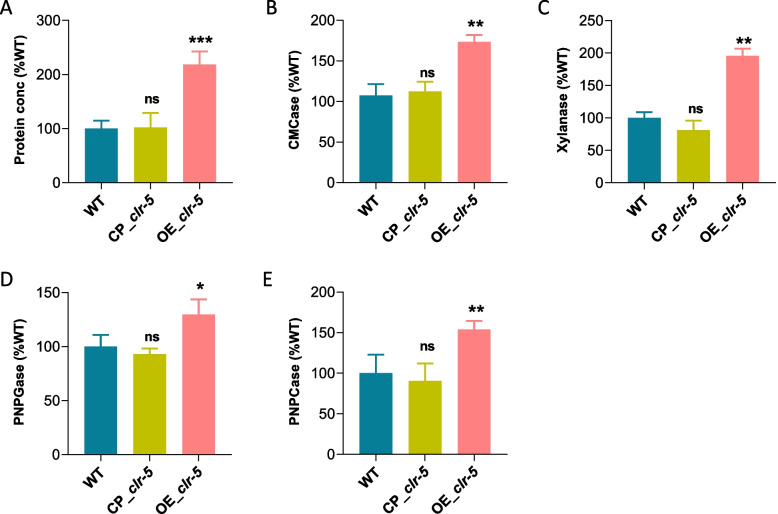
Phenotypic analysis of *N. crassa* strains CP_*clr-5* and OE_*clr-5* on 2% Avicel. The assessment of extracellular protein concentration (**A**) and the enzymatic activities of endo-glucanase (**B**), xylanase (**C**), β-glucosidase (**D**), and exo-cellulase (**E**) in the supernatants from cultures of *N. crassa* strains CP_*clr-5* and OE_*clr-5* after 7 days of growth on Avicel, in comparison to the WT strain. *, **, and *** represent significant difference at *p* < 0.05,* p* < 0.01, and *p* < 0.001, respectively; ns represents not significant. Error bars indicate the standard deviations from at least three biological replicates

To further confirm the role of Clr-5 in cellulase production, we generated a *clr-5*-complemented strain (CP_*clr-5*) in the Δ*clr-5* background, and an overexpression strain (OE_ *clr-5*) in the WT strain background. When cultivated on Avicel, CP_*clr-5* exhibited the restoration of secreted proteins and lignocellulolytic enzyme activities to levels similar to those of the WT strain. Meanwhile, OE_*clr-5* exhibited significant significant enhancements in secreted proteins (~ 118%), endo-glucanase activity (~ 73%), xylanase activity (~ 95%), β-glucosidase activity (~ 30%), and exocellulase activity (~ 54%), compared with those of the WT strain after growth on Avicel (Fig. 2A–E). These findings strongly support the positive regulatory role of Clr-5 in cellulase production and the growth rate of *N. crassa* on Avicel.

### Transcriptomic analysis of *N. crassa* with the deletion of *clr-5* on cellulose

**Fig. 3:**
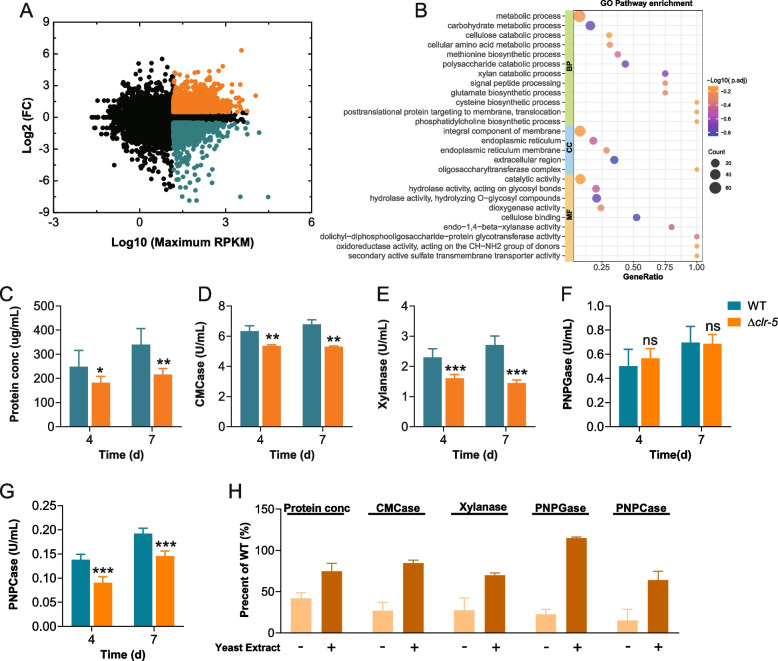
Transcriptomic profiles of *N. crassa* Δ*clr-5* on Avicel. **A** Differential expression analysis of ∆*clr-5* compared with WT strain when induced by 2% Avicel for 4 h. Log2 ratio of *∆clr-5*/WT *vs.* Log10 maximum RPKM in either strain. Up-regulated genes are shown in orange; down-regulated genes are shown in dark cyan. Detailed data are provided in Additional file 2: Table S3. **B** GO analysis of 371 genes with down-regulated expression in strain ∆*clr-5*. Total extracellular protein concentration (**C**), endo-glucanase activity (**D**), xylanase activity (**E**), β-glucosidase activity (**F**), and exocellulase activity (**G**) of cultures of *N. crassa* grown in Avicel medium supplemented with 0.75 g/L yeast extract. **H** Comparison of total extracellular protein concentration and lignocellulolytic enzyme activities of ∆*clr-5* relative to that of the WT strain after 4 d of culture in Avicel medium with or without 0.75 g/L yeast extract. Error bars in (**C-D**) indicate the standard deviations from at least three biological replicates

To explore the mechanism by which Clr-5 regulates cellulase production, we performed a transcriptional profile analysis of the WT and Δ*clr-5* strains cultured on Avicel. Compared with the WT strain, Δ*clr-5* showed significantly altered transcript levels of 976 genes, with 371 genes being significantly down-regulated and 605 genes being significantly up-regulated (Fig. 3A and Additional file 2: Table S3). A Gene Ontology (GO) analysis of the down-regulated genes in Δ*clr-5* revealed significant enrichment of genes involved in the polysaccharide catabolic process, xylan catabolic process, and cellulose catabolic process, consistent with the phenotypes observed in Δ*clr-5* on Avicel (Fig. 3B and Additional file 2: Table S4). The analysis of the transcriptional profiles revealed that 47 genes encoding Carbohydrate-Active Enzymes (CAZymes) showed significantly lower transcript levels in Δ*clr-5* than in the WT strain, These genes included cellulase genes (*gh61-1*, *gh61-3*, *gh61-5*, *gh61-6*, *gh5-1*, *gh6-3*, *gh7-1*, *gh7-2*, and *gh7-4*) and hemicellulase genes (*gh10-1*, *gh10-2*, *gh10-3*, *gh11-1*, *gh11-2*, *gh43-6*, *gh51-1*, and *gh53-1*). In addition to these genes encoding CAZymes, genes related to protein synthesis and secretion were also significantly down-regulated in Δ*clr-5*, including those encoding the protein disulfide-isomerase PdiA (NCU09223), two ER chaperones (HSP70-6, NCU09485 and CNX-1, NCU09265), and four translocation proteins SEC61(NCU04127 and NCU08379), SEC62 (NCU06333), and SEC63 (NCU00169) [26, 41].

Previous studies have demonstrated the regulatory crosstalk between nitrogen and carbon metabolism during plant biomass utilization by filamentous fungi. For example, in *A. nidulans*, the expression of cellulase genes is affected by AreA, a global nitrogen transcription factor [42]. In *N. crassa*, the genes encoding proteins involved in amino acid metabolism are included in the Avicel regulon and the CLR-1/CLR-2 regulon [16]. An analysis of transcriptomic data indicated that these down-regulated genes in Δ*clr-5* were enriched in the functional categories of cellular amino acid metabolic process, including the biosynthesis of methionine, glutamate, and cysteine, and the degradation of histidine and tryptophan (Fig. 3B and Additional file 2: Table S4), suggesting that Clr-5 may be essential for amino acid metabolism. Consequently, we speculated that amino acid metabolism plays an important role in the expression of genes encoding cellulases in *N. crassa*.

To test this hypothesis, we tested the secreted proteins and (hemi)cellulolytic enzyme activities of the WT and Δ*clr-5* strains in Avicel medium supplemented with yeast extract. Yeast extract is a rich nitrogen source, which might partially alleviate the effects of impaired nitrogen metabolism. As shown in Fig. 3C–H, the incorporation of yeast extract significantly mitigated the reduction in both cellulase and hemicellulase activities of Δ*clr-5*: secreted proteins (from ~ 42% to ~ 75% of the WT strain), endo-glucanase activity (from ~ 27% to ~ 85% of the WT strain), xylanase activity (from ~ 28% to ~ 70% of the WT strain), β-glucosidase activity (from ~ 23% to ~ 115% of the WT strain), and exocellulase activity (from ~ 37% to ~ 70% of the WT strain) after culture for 4 d on Avicel. Composite nitrogen sources, including peptone and yeast extract, are abundant in amino acids, peptides, and other vital nutrients. These sources provide vital components that alleviate metabolic deficiencies, thereby differentiating between metabolic impairment and the shortcomings of cellulase synthesis. These results further highlight the significance of nitrogen metabolism in cellulose degradation and suggest that Clr-5 may affect cellulose degradation by regulating nitrogen metabolism.

### Clr-5 regulates amino acid metabolism in *N. crassa*

**Fig. 4:**
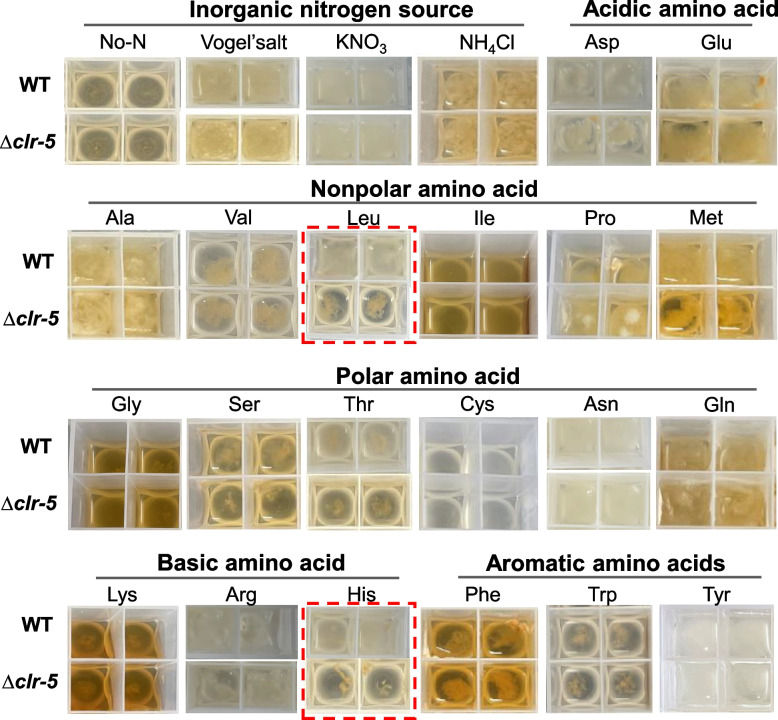
Growth of WT and *clr-5* mutant in liquid media with different nitrogen sources. Cells were inoculated into 3 mL liquid medium containing 2% glucose and indicated nitrogen source in round-bottomed 24-well plates and incubated at 25 °C (nitrogen concentration, 50 mM). Pictures were taken after 96 h of culture. Representative image of at least 3 biological replicates

**Fig. 5:**
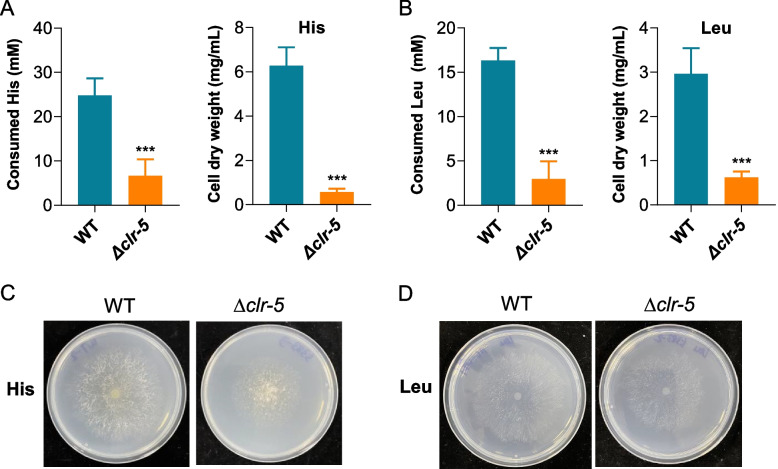
Physiological characterizations of *N. crassa* with leucine or histidine as sole nitrogen source. Consumption of amino acid and cell dry weight of WT and ∆*clr-5* when grown in glucose media with histidine (**A**) or leucine (**B**) as the sole nitrogen source for 96 h and 48 h, respectively, in round-bottomed 24-well plates. Growth profile of strain ∆*clr-5* on agar plate supplemented with histidine (**C**) or leucine (**D**) after 60 h of incubation at 25 °C. Error bars in (**A-B**) indicate the standard deviations from at least three biological replicates

Several transcription factors involved nitrogen metabolism have been identified and characterized in *N. crassa*, including Nit-2, Nit-4, Amn-1, Pco-1 [43]. To explore the role of Clr-5 in amino acid metabolism, we assessed the growth phenotypes of the WT and Δ*clr-5* strains on media containing individual amino acids and inorganic nitrogen sources (nitrate and ammonium) as the sole nitrogen source, using glucose as the carbon source to exclude the effect of cellulose degradation. As shown in Fig. 4, *N. crassa* could grow with all the tested nitrogen sources, except cysteine. Δ*clr-5* exhibited growth defects on methionine, serine, threonine, leucine, and histidine, with leucine and histidine causing the most severe defects. When leucine or histidine was supplied as the sole nitrogen source, the mycelia of Δ*clr-5* clumped together and grew slower than did the WT strain (Fig. 4). After 4 days of growth in medium containing histidine or leucine as the sole nitrogen source, Δ*clr-5* exhibited a 90.9% and 79% reduction in cell dry weight, respectively, compared to the WT strain. Additionally, the consumption rates of histidine and leucine in Δ*clr-5* were also reduced by 73% and 79%, respectively, compared with those in the WT strain (Fig. 5A-B). Consistently, the deletion of *clr-5* led to reduced radial growth on agar plate with histidine or leucine as the sole nitrogen source (Fig. 5C-D).

**Fig. 6:**
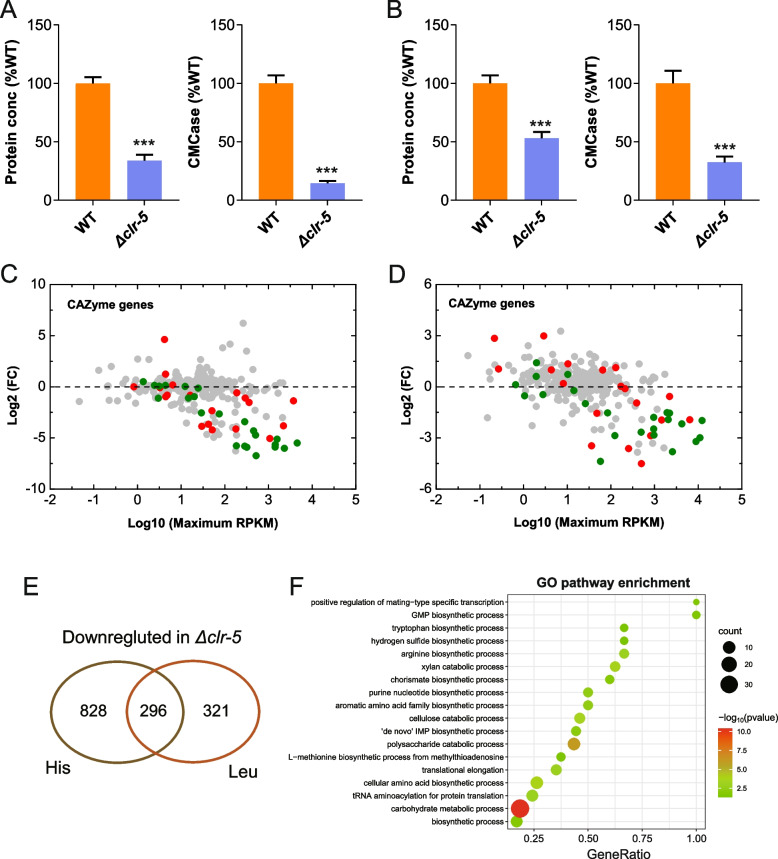
Phenotypic analysis of *clr-5* mutant in Avicel media with histidine or leucine as sole nitrogen source. Secreted protein and endo-glucanase activity of WT and ∆*clr-5* after 7 d of culture on Avicel with histidine (**A**) or leucine (**B**) as the sole nitrogen source. Relative mRNA abundance of all CAZyme genes in Δ*clr-5* mutant *vs.* WT when grown on cellulose with histidine (**C**) or leucine (**D**) as the sole nitrogen source. Cellulase genes are shown in olive, hemicellulase genes are shown in red. **E** Venn diagram of genes with significantly reduced transcript levels in strain ∆*clr-5* relative to WT in response to histidine and leucine. **F** Enrichment of 296 genes with down-regulated expression in strain ∆*clr-5* within the Biological Process (BP) category in GO analysis. Values and error bars represent means and standard deviations of independent triplicate experiments, respectively. Error bars in (**A-B**) indicate the standard deviations from at least three biological replicates

Subsequently, we measured the secreted protein and cellulase activities of the WT and Δ*clr-5* strains when cultured in Avicel medium with histidine or leucine as the sole nitrogen source. As expected, the protein concentration and cellulolytic enzyme activities were remarkably reduced in Δ*clr-5* compared to the WT strain (Fig. 6A–B). We then conducted a transcriptional profile analysis of the WT and Δ*clr-5* strians cultured in Avicel medium with leucine or histidine as the sole nitrogen source. Expression pattern analyses revealed that, compared with the WT strain, Δ*clr-5* showed significant down-regulation of 1123 and 617 genes in response to histidine and leucine, respectively (Fig. 6E and Additional file 2: Table S6). Functional enrichment analysis using the GO database revealed that the set of 396 overlapping genes was mainly enriched in functional categories of ‘carbohydrate metabolism’ and ‘amino acid metabolism’ (Fig. 6F and Additional file 2: Table S7). These overlapping set of genes included several involved in amino acid metabolism, such as those related to histidine metabolism (NCU11365, aminotransferase; NCU09320, ATP phosphoribosyltransferase; His-4, histidinol-phosphatase), branched-chain amino acids metabolism (NCU06881, aminotransferase; NCU03607, 3-hydroxyaspartate dehydratase; Leu-4, 2-isopropylmalate synthase; and NCU00792, amino acid aminotransferase) and aromatic amino acid metabolism (Additional file 2: Table S4). Moreover, a gene encoding glutamate dehydrogenase (NCU01195, *am-1*), which catalyzes the synthesis of glutamate from ammonium and α-oxoglutarate, was also down-regulated in strain Δ*clr-5*. In *N. crassa* and *A. nidulans*, disruption of *am-1* results in significantly poor growth with ammonium as the sole nitrogen source [44, 45].

Consistent with the growth defects on serine and threonine, Δ*clr-5* also showed significantly reduced induction of the genes related to the metabolism of serine and threonine, including NCU08409, *oxD*, *thr-4*, NCU03607, *ser-2*, *hom-1*. Additionally, genes encoding amino acid permeases, such as *pmg*, NCU00711, and NCU09458, were down-regulated in Δ*clr-5*. Transcriptomic analysis indicated that the transcript levels of genes encoding CAZymes were significantly lower in Δ*clr-5* than in the WT strain, consistent with the significant reduction in protein and cellulolytic enzyme activities in Δ*clr-5* (Fig. 6 and Additional file 2: Table S5). These down-regulated genes encoded CAZymes in the glycoside hydrolase family (GH1, GH3, GH43, GH6 and GH7), cellobiose dehydrogenases (AA8, CDH), and lytic polysaccharide monooxygenases (AA9, LPMO). When grown on histidine, 16 out of a total of 23 cellulase genes and 13 out of 16 hemicellulase genes showed significantly decreased transcript levels in Δ*clr-5* (Fig. 6C). Similarly, 16 genes encoding cellulases and nine genes encoding hemicellulases were significantly down-regulated in Δ*clr-5* when it was grown with leucine as the sole nitrogen source (Fig. 6D). Moreover, there was a significant decrease in transcript levels of genes encoding transcriptional factors in Δ*clr-5*, including Clr-1, Clr-2, Clr-4, and TRC-1, all of which positively regulate the expression of genes encoding lignocellulolytic enzymes [16, 27, 38]. Taken together, these results demonstrate that Clr-5 is essential for amino acid metabolism, and that to some extent Clr-5 regulates cellulase gene expression by controlling amino acid metabolism.

### Disruption of *clr-5* results in reduced stress tolerance

Previous studies have shown that the transcription factors associated with the regulation of cellulase genes can also contribute to the response to external stress responses in filamentous fungi, such as Clr-1, Clr-2 and ClrC [16, 43]. To investigate whether Clr-5 is involved in regulating the response to a range of stresses, including cell wall disruption and high osmolality, we assessed the sensitivity of the WT and Δ*clr-5* strains to Calcofluor White, Congo Red, and NaCl. The growth of Δ*clr-5* was reduced by 10%, compared to WT when grown on agar plates containing 100 μg/mL Calcofluor White (Additional file 1: Fig. S2,), which disrupts cell wall synthesis by binding to chitin [46]. Additionally, Δ*clr-5* also showed lower resistance to 2 mg/mL Congo Red, which binds to β-1,3-glucans and interferes with the cell wall [47]. Those data suggested that Clr-5 plays an important role in maintaining cell wall integrity. When Δ*clr-5* was cultivated on agar plates containing 1 M NaCl, its colonies were smaller than those of the WT strain, indicating that Clr-5 is also involved in osmotic stress tolerance.

### The function of Clr-5 is conserved in ascomycete

**Fig. 7:**
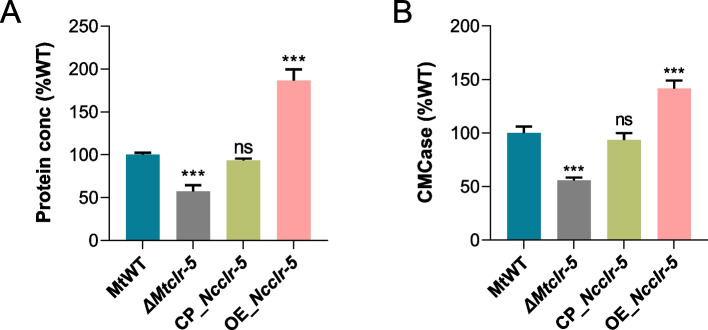
Protein production and enzyme activity phenotypes of *M. thermophila* strains. MtWT, *M. thermophila* wild-type strain; ∆*Mtclr-5*, mutant of *Mtclr-5*; CP_*Ncclr-5*, complementation strain of mutant Δ*Mtclr-5* with *clr-5* from *N. crassa*; OE_*Ncclr-1*, Overexpression of *N. crassa clr-5* in the *M. thermophila* WT background. Total secreted protein (**A**) and endo-glucanase activity (**B**) in the supernatants of cultures after 3 d of the culture on 2% Avicel. Error bars in indicate the standard deviations from at least three biological replicates

To assess whether the function of Clr-5 is conserved in filamentous fungi, we characterized the mutant of its ortholog (Mycth_2301131, named MtClr-5) in the thermophilic cellulolytic fungus *M. thermophila. M. thermophila* is another biomass-degrading organism that has been used as a model system to investigate both basic fungal cell biology, such as the mechanism of protein secretion, and for industrial fungal engineering to produce biochemicals directly from plant biomass [9, 10], based on the suite of available molecular biology tools, including genome-editing techniques [18]. Our findings revealed that Δ*Mtclr-5* similarly exhibited decreased protein secretion (~ 56%) and endo-glucanase activity (~ 44%) compared with those of *M. thermophila* wild-type strain (MtWT) after 3 d of growth in Avicel medium (Fig. 7). This result confirms the important role of MtClr-5 in cellulase production in *M. thermophila*, mirroring the function of its ortholog in *N. crassa*.

To further investigate and compare the conserved function of Clr-5 in cellulose degradation between the two fungi, we complemented the mutant Δ*Mtclr-5* with the open reading frame of *clr-5* from *N. crassa* under the control of the native promoter *Mtclr-5*. Additionally, we overexpressed *clr-5* from *N. crassa* in the MtWT strain under the control of the strong constitutive promoter *MtgpdA* (MYCTH_2298136). Those complemented and overexpression strains were designated as CP_*Ncclr-5* and OE_*Ncclr-5*, respectively. Interestingly, the protein secretion and enzyme activities in the interspecies complementation strain CP_*Ncclr-5* were restored to the levels similar to those of the MtWT strain when grown on Avicel. Furthermore, the OE_ *Ncclr-5* strain exhibited a significant increase in secreted proteins (~ 187%) and endo-glucanase activity (~ 142%), compared with those of the MtWT strain after 3 days of growth on Avicel (Fig. 7). These results confirm that the function of Clr-5 is critical for cellulose degradation and is evolutionarily conserved in ascomycete fungi.

## Conclusions

In this study, we identified an Zn_2_Cys_6_ transcription factor Clr-5 in *N. crassa*, which regulates the expression of genes encoding cellulases when grown on cellulose. Compared with the WT strain, Δ*clr-5* exhibited a significant decrease in secreted protein (~ 54%), endo-glucanase activity (~ 45%), xylanase activity (~ 67%), β-glucosidase activity (~ 62%), and exocellulase activity (~ 60%). Comparative transcriptomic analyses indicated that Clr-5 is essential for cellulase production and amino acid metabolism. Our comparative transcriptomic analyses illuminated the pivotal roles of Clr-5, highlighting its indispensability in both cellulase production and amino acid metabolism. Notably, when leucine or histidine served as the sole nitrogen source, Δclr-5 exhibited severe growth impairments on both glucose and Avicel media. Furthermore, the expression of most genes encoding CAZymes and those involved in amino acid uptake and the metabolism of histidine, branched-chain amino acids, and aromatic amino acids, were down-regulated in strain Δ*clr-5* when grown in Avicel medium supplemented with histidine or leucine as the sole nitrogen source. Significantly, our findings also underscore the evolutionary conservation of Clr-5 function across ascomycete fungi.

## Materials and methods

### Strains and culture conditions

*Neurospora crassa* wild-type strain (WT, FGSC #2489) and NCU05383 mutant (FGSC #11,019) were obtained from the Fungal Genetics Stock Centre (FGSC). *N. crassa* strains were pre-cultured in 1 × Vogel’s minimal medium (VMM) with 2% (w/v) sucrose. The cultures were incubated in the dark at 28° C for 1–2 d, followed by 6–8 d under constant light at room temperature to produce mature conidia.

*M. thermophila* wild-type strain (MtWT, ATCC 42464) and its mutants were grown on 1 × VMM supplemented with 2% (w/v) glucose at 35 °C to obtain mature conidia. Antibiotic was added when needed to screen for transformants.

*Escherichia coli* March-T1 was used for vector manipulation and cultivated in Luria–Bertani (LB) medium with 100 µg/mL ampicillin or 50 µg/mL kanamycin for plasmid selection.

### Plasmid and strain construction

All primer sequences used in this study are listed in Additional file 2: Table S1. To rescue *clr-5*, the upstream region (1028 bp) and the full-length NCU05383 sequence (3067 bp) were amplified from *N. crassa* genomic DNA and ligated into plasmids pAN52-bar [27] digested with *Bgl*II and *Bam*HI using the Gibson kit to form the complementation plasmids pAN52-CP-clr5. Complementary plasmid was linearized and transformed into *N.crassa* Δ*clr-5* and the transformants were screened by selection for phosphinothricin resistance. To overexpress *clr-5*, the ORF region of *clr-5* under the control of the strong constitutive *tef1* (NCU02003) promoter of *N. crassa* was inserted into between *Bgl*II and *Bam*HI sites of plasmid pAN52-bar, generating the plasmid pAN52-OE-clr-5. The constructed recombinant plasmid linearized were separately transformed into strain Δ*clr-5* and the transformants were screened by selection for phosphinothricin resistance [6]. The presence of the transgenes was confirmed by PCR.

To delete *Mtclr-5* in *M. thermophila*, the gene coding region was replaced by the expression cassette of marker gene *neo*. This process needs three DNA fragments: Cas9, sgRNA, and Donor fragments. Cas9-expressing cassette was amplified from plasmid p0380-bar-P*tef1*-Cas9-TtprC [48] using the primer pairs (Additional file 2: Table S1). The target sites of genes were designed by sgRNACas9 tool [48]. The sgRNA was constructed by connecting U6 promoter, genes target sites and gRNA. The 5’ and 3’ flanking fragments of *Mtclr-5* were separately amplified from *M. thermophile* genomic DNA. The resultant PCR fragments as well as the selectable marker cassette PtrpC-neo from plasmid p0380-neo [49] were assembled and inserted into pPK2BarGFP plasmid digested by XbaI/EcoRV using the Gibson Assembly Cloning Kit, to generate donor DNA sequence donor-Mtclr-5-neo. To complement the mutant Δ*Mtclr-5* with *clr-5* from *N. crassa*, the open reading frame of *clr-5* was amplified from *N. crassa* genomic DNA and cloned into plamsid pAN52-bar under the control of the native promoter of *Mtclr-5* using the the Gibson kit, to form the complementation plasmid pAN52-CP-Ncclr-5. For the construction of plasmids overexpressing *N.crassa clr-5* in *M. thermophila*, the fragment of *N. crassa clr-5* and the strong constitutive promoter of *MtgpdA* (MYCTH_2298136) were assembled and inserted into plasmid pAN52-bar digested by *Bgl*II/*Bam*HI using the Gibson Assembly Cloning Kit, to generate vector pAN52-OE-Ncclr-5.

PEG-mediated transformation of *M. thermophila* protoplasts was performed as described previously [48]. For the deletion of *Mtclr-1*, a mixture of 10 μg PCR amplicons of the Cas9-expression cassette, donor DNA cassette, and sgRNA cassette at a molar concentration ratio of 1:1:1 was cotransformed into *M. thermophila* protoplasts. For gene overexpression, 10 µg linearized plasmids pAN52-CP-Ncclr-5 and pAN52-OE-Ncclr-5 were transformed into the protoplasts of the *M. thermophila* Δ*Mtclr-5* and the wild-type strain to generate strains CP_Ncclr-5 and OE_Ncclr-5, respectively. Putative transformants were selected with corresponding antibiotics, followed by verification via PCR with paired primers (Additional file 2: Table S1).

### Quantification of secreted proteins and enzyme activity

To quantify secreted proteins and enzyme activity, each *N. crassa* strain was inoculated into 100 mL 1 × VMM supplemented with 2% w/v Avicel in a 250-mL Erlenmeyer flask and incubated at 25 °C with shaking at 200 rpm on a rotary shaker. Samples were collected at specified intervals for analysis.

The total extracellular protein in the culture supernatant was quantified using a Bio-Rad protein assay kit (Bio-Rad, Hercules, CA, USA). The activities of endoglucanase and endo-1,4-β-xylanase in the culture supernatant were determined using an Azo-CM-Cellulose assay kit (Megazyme, Wicklow, Ireland) and an Azo-Xylan kit (Megazyme), respectively. The activity of β-glucosidase was determined using *p*-nitrophenyl-β-d-glucopyranoside (*p*NPG) as the substrate. The reaction mixture contained 250 μL diluted enzyme and 250 μL substrate (1.0 mg/mL) in 50 mM citrate buffer (pH 4.8), and was incubated for 10 min at 50 °C. The reaction was terminated by adding 500 µL 1 M Na_2_CO_3_ and the amount of *p*-nitrophenol (*p*NP) released was calculated based on the absorbance at 420 nm. A standard curve was constructed using known concentrations of *p*NP. Exoglucanase (PNPCase) activity in the culture supernatant was determined using *p*-nitrophenyl β-D-cellobioside as the substrate. In the exoglucanase and β-glucosidase activity analyses, one unit (U) of enzymatic activity was defined as the amount of *p*NP released by 1 mL enzyme from the substrate per minute.

### Cell dry weight measurement

To determine the dry weight of the cell biomass in Avicel medium, the fungal mycelia were harvested after incubation for 4 d and 7 d. A 10-mL aliquot of thoroughly mixed culture broth was centrifuged at 4,000 g for 10 min. After discarding the supernatant, 6 mL acetic-nitric reagent, comprising acetic acid, nitric acid, and water in a molecular ratio of 8:1:2, was added and the mixture was boiled in water for 2 h to solubilize the fungal cells. The reaction mixture containing the residual Avicel was then centrifuged, dried, and weighed. The acetic nitric reagent was prepared by mixing 150 mL 80% acetic acid with 15 mL concentrated nitric acid.

### The analysis of transcriptomic data

The *N. crassa* strains WT and Δ*clr-5* were pre-cultured in 100 mL of 1 × Vogel’s salt with 2% glucose for 16 h in constant light with constant shaking at 200 rpm at 25 °C. The mycelia were then washed three times with 1 × VMM without carbon source (for a transfer to VMM with 2% Avicel) or 1 × VMM without a nitrogen source (for a transfer to VMM without a nitrogen source, supplemented with 2% Avicel as carbon source and 50 mM histidine or leucine as the sole nitrogen source). The mycelia were then resuspended in 100 mL indicated medium and grown as described above for 4 h. After culturing, mycelia were harvested and immediately frozen in liquid nitrogen. Total RNA was extracted using the TRIzol reagent (Invitrogen, Carlsbad, CA, USA) [36] and contaminating DNA was removed using the RNeasy DNase I kit (Qiagen, Hilden, Germany).

The cDNA libraries were prepared using the standard protocols of Illumina (San Diego, CA, USA) and sequenced on the Illumina Novaseq platform to generate 150 bp paired-end reads. Independent duplicate cultures were sampled to avoid random errors. The RNA sequences were aligned to the predicted transcripts from the *N. crassa* OR74A genome (v12) [50] using Tophat (v2.0.12) [51]. The number of reads uniquely mapping to only one gene was calculated for each gene by HTSeq-count (http://www-huber.embl.de/users/anders/ HTSeq) using SAM files and genome annotation files as inputs. Normalized expression values (RPKM, Reads Per Kilobase per Million mapped reads) for each gene were calculated from the number of uniquely mapped reads (Additional file 2: Table S2). Differentially expressed genes were identified using the DESeq package (v1.5.1) [52] with the number of raw reads mapped to unique genes as the inputs. Unless otherwise noted, genes with |log2 FoldChange|≥ 0.5 and DESeq *P*-adj value < 0.05 were considered to be significantly differentially expressed between two samples. The raw reads of transcriptomic data have been deposited in Gene Expression Omnibus (GEO, accession number: GSE222372) at the National Center for Biotechnology Information (NCBI).

### Growth and physiological characteristics of *N. crassa* on various nitrogen sources

In these experiments, Vogel’s salts were prepared without ammonium nitrate, and nitrogen sources were added as described. Each of *N. crassa* strains was inoculated into 3 mL medium containing individual amino acids and inorganic nitrogen sources (nitrate and ammonium) and 2% w/v glucose. The nitrogen concentration was fixed at 50 mM. Cultures were photographed after 48 h (when using KNO_3_, NH_4_Cl, aspartic acid, glutamic acid, alanine, leucine, proline, methionine, glutamine, arginine, or tyrosine as the sole nitrogen source) or 96 h (when employing valine, isoleucine, glycine, serine, threonine, cysteine, asparagine, lysine, histidine, phenylalanine, or tryptophan as the sole nitrogen source) at 25 °C. The contents of histidine and leucine were determined by liquid chromatography-tandem mass spectrometer (LC–MS/MS) [10]. To assess the growth patterns of the ∆clr-5 strain on agar plates supplemented with histidine or leucine, photographs were taken after 60 h of incubation at 25 °C.

### The analysis of the tolerance to chemical and environmental stresses

To evaluate the sensitivity of fungal strains to oxidative stress, osmotic stress, and cell wall disturbance, 3 μL aliquots of conidial suspension (10^5^) were applied to Vogel’s solid medium (1 × Vogel’s salts, 2% w/v sucrose, and 1.5% w/v agar) supplemented with NaCl (1.0 M), Congo Red (2 mg/mL), and Calcofluor White (100 μg/mL), respectively. The plates were incubated at 28 °C for 25 h. The diameter of colonies was measured to calculate growth indices under each stress.

### Statistical significance tests

One-tailed homoscedastic (equal variance) *t*-test was used for adjusting statistical significance. In figures and tables, n.s. indicates no statistical significance; and *, **, and *** represent significant difference at *p* < 0.05, *p* < 0.01, and *p* < 0.001, respectively.

### Supplementary Information

**Additional file 1: Fig. S1. **Biomass accumulation of *N. crassa* strains WT and Δ*clr-5* grown in 1×VMM with 2% glucose for 48 h. Error bars indicate the standard deviations from at least three biological replicates. **Fig. S2. **The sensitivities of the strain Δ*clr-5* to high osmotic stress and cell wall disturbance. The mature spore was inoculated onto agar plates alone or supplemented with 100 μg/mL Calcofluor White, 2 mg/mL Congo Red, and 1 M Nacl and incubated at 25 °C for 25 h. The values and error bars represent means and standard deviations of independent triplicate experiments, respectively. Error bars indicate the standard deviations from at least three biological replicates. **Fig. S3. **Raw gels image of Fig. 1G. SDS-PAGE analysis of the proteins secreted by* N. crassa *strains WT and *Δclr-5* grown in Avicel medium for 4 d and 7 d.

**Additional file 2: Table S1.** List of PCR primers used in this study. **Table S2.** The profiles of RNA-seq reads mapped to the genome of* N. crassa* and the differential expression analysis. **Table S3.** Genes showing significantly differential transcriptional levels in strain ∆clr-5 compared to the wild-type strain in 1×VMM with 2% Avicel. **Table S4.** GO analysis of the 371 genes with down-regulated expression levels in strain ∆clr-5 in 1×VMM with 2% Avicel. **Table S5.**Transcriptional levels of CAZyme genes in strain Δclr-5 when grown in Avicel medium with histidine or leucine as the sole nitrogen source. **Table S6.** The genes with significantly reduced transcript levels in strain ∆clr-5 relative to WT in response to histidine and leucine. **Table S7.** GO analysis of the 296 genes with down-regulated expression levels in strain ∆clr-5 relative to WT in response to histidine and leucine.

## Data Availability

All data generated or analyzed during this study are included in this published. article and its supplementary information files.

## Abbreviations

CAZyme: Carbohydrate-active enzyme
CDH: Cellobiose dehydrogenases
Clr-5: Cellulose degradation regulator 5
GH: Glycoside hydrolase family
RNA-seq: RNA-sequencing
RPKM: Reads Per Kilobase per Million mapped reads
