# Supplementary material for: The transcriptional factor Clr-5 is involved in cellulose degradation through regulation of amino acid metabolism in Neurospora crassa
Source: BMC Biotechnol. 2023 Nov 29;23:50. doi: 10.1186/s12896-023-00823-4 (PMC10687990; doi:10.1186/s12896-023-00823-4)
Supplement: Supplementary file 1 — Additional file 1: Fig. S1. Biomass accumulation of N. crassa strains WT and Δclr-5 grown in 1×VMM with 2% glucose for 48 h. Error bars indicate the standard deviations from at least three biological replicates. Fig. S2. The sensitivities of the strain Δclr-5 to high osmotic stress and cell wall disturbance. The mature spore was inoculated onto agar plates alone or supplemented with 100 μg/mL Calcofluor White, 2 mg/mL Congo Red, and 1 M Nacl and incubated at 25 °C for 25 h. The values and error bars represent means and standard deviations of independent triplicate experiments, respectively. Error bars indicate the standard deviations from at least three biological replicates. Fig. S3. Raw gels image of Fig. 1G. SDS-PAGE analysis of the proteins secreted by N. crassa strains WT and Δclr-5 grown in Avicel medium for 4 d and 7 d. [file 12896_2023_823_MOESM1_ESM.docx]

**Fig. S1** Biomass accumulation of *N. crassa* strains WT and Δ*clr-5* grown in 1×VMM with 2% glucose for 48 h. Error bars indicate the standard deviations from at least three biological replicates.


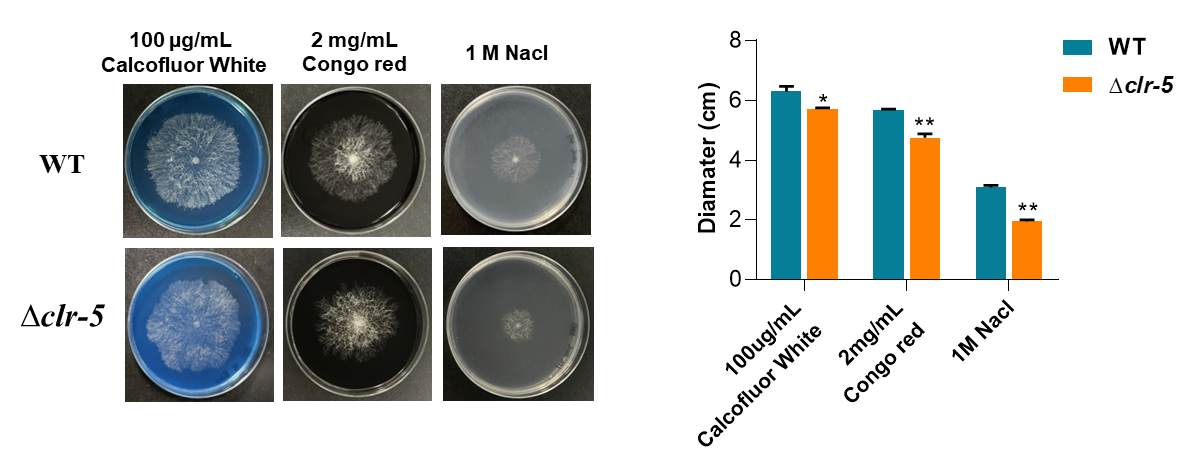


**Fig. S2** The sensitivities of the strain Δ*clr-5* to high osmotic stress and cell wall disturbance. The mature spore was inoculated onto agar plates alone or supplemented with 100 μg/mL Calcofluor White, 2 mg/mL Congo Red, and 1 M Nacl and incubated at 25 °C for 25 h. The values and error bars represent means and standard deviations of independent triplicate experiments, respectively. Error bars indicate the standard deviations from at least three biological replicates.


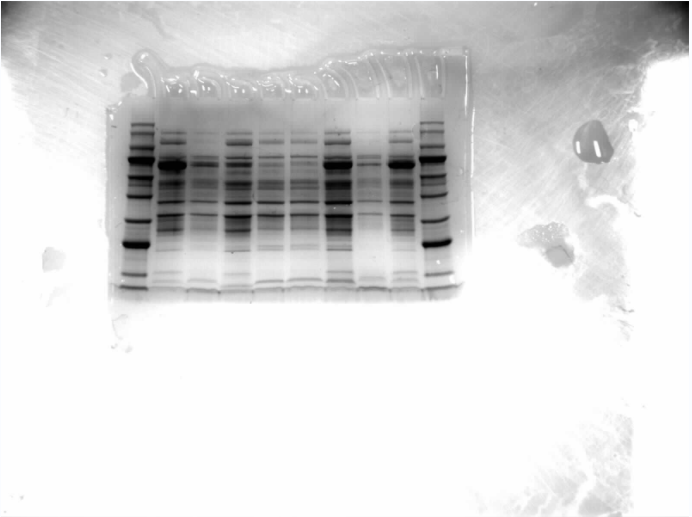


**135−**

**63−**

**48−**

**35−**

**25−**

**17−**

**180−**

**75−**

**100−**

**∆*clr-5*-4d**

**WT-7d**

**∆*clr-5*-7d**

**M**

**WT-4d**

**Fig. S3** Raw gels image of Fig. 1G. SDS-PAGE analysis of the proteins secreted by *N. crassa* strains WT and *Δclr-5* grown in Avicel medium for 4 d and 7 d.
